# Editorial: Insights in non-neuronal cells: 2022

**DOI:** 10.3389/fncel.2023.1332869

**Published:** 2023-11-28

**Authors:** Jessica M. Rosin, Marie-Ève Tremblay, Jason R. Plemel

**Affiliations:** ^1^Department of Oral Biological and Medical Sciences, University of British Columbia, Vancouver, BC, Canada; ^2^Life Sciences Institute, University of British Columbia, Vancouver, BC, Canada; ^3^Division of Medical Sciences, University of Victoria, Victoria, BC, Canada; ^4^Institute on Aging and Lifelong Health, University of Victoria, Victoria, BC, Canada; ^5^Center for Advanced Materials and Related Technology, University of Victoria, Victoria, BC, Canada; ^6^Neurosciences Axis, Centre de Recherche du CHU de Québec, Université Laval, Québec City, QC, Canada; ^7^Department of Molecular Medicine, Université Laval, Québec City, QC, Canada; ^8^Department of Neurology and Neurosurgery, McGill University, Montréal, QC, Canada; ^9^Department of Biochemistry and Molecular Biology, University of British Columbia, Vancouver, BC, Canada; ^10^Neuroscience and Mental Health Institute, University of Alberta, Edmonton, AB, Canada; ^11^Department of Medicine, Division of Neurology, University of Alberta, Edmonton, AB, Canada; ^12^Department of Medical Microbiology and Immunology, University of Alberta, Edmonton, AB, Canada

**Keywords:** microglia, radial glia cells, tanycytes, neural stem and precursor cells (NPCs), astrocytes, oligodendrocyte, oligodendrocyte progenitor cell

As we embark upon the third decade of the 21st Century, the scientific community has witnessed remarkable achievements in recent years, particularly in the domain of non-neuronal cells. These include resident immune cells, glial cells (such as astrocytes and cells of the oligodendrocyte lineage), neurovascular cells (including endothelial cells, pericytes, perivascular macrophages, and more), as well as other cell types inhabiting the nervous system. In light of these advancements, Frontiers has orchestrated a series of Research Topics to showcase the latest breakthroughs in various research fields.

This special edition of our Research Topic aims to provide insights into the progress achieved over the past decade within the non-neuronal cells field, while also addressing the challenges that lie ahead. Our editorial initiative is centered on presenting new perspectives, emerging developments, ongoing obstacles, recent discoveries, and future outlooks in the realm of non-neuronal cells. We aspire to both inspire and inform researchers, offering them valuable direction and guidance. This special edition comprises six distinct articles that delve into these topics.

Microglia are critical regulators of brain development, but only more recent work focuses on the importance of neuronal subtype in microglia-neuron interactions. In a Perspective article by Ngozi and Bolton they discuss what is known about how neuronal subtype impacts the interactions between microglia and neurons in the developing brain, and in particular, how subpopulations of microglia often treat excitatory and inhibitory neurons differently. The developing hypothalamus is highlighted, and the authors discuss their recent work showing that even the type of neuropeptide produced by a neuron can impact microglial behaviors. This emerging field holds the potential for future therapies to target unique microglia-neuron interactions, which may offer new strategies for personalized medicine.

Two Mini Reviews next provide novel insights into the regulation of microglial function by neural stem cells, and the functional relationship between embryonic radial glia and tanycytes during development. de Almeida et al. discuss the involvement of neural stem and precursor cells (NPCs) as regulators of microglial biology, including microglial survival, proliferation, migration, phagocytosis, and reactivity. These immunomodulatory roles are described in the developing, injured, and degenerating central nervous system (CNS), highlighting the importance of these findings to future therapies aimed at targeting CNS regeneration and repair in cases of traumatic brain insults and neurodegeneration.

Fong and Kurrasch provide a comprehensive review of tanycyte biology in the hypothalamus, beginning with the development of these cells from embryonic radial glia to their emerging roles in the neural stem cell niche postnatally. Similar to hypothalamic radial glia, tanycytes line the third ventricle and extend a long process into the hypothalamic parenchyma. This heterogeneous population of cells contribute to a broad range of physiologies, including energy balance, which places them in a unique position to regulate neural stem cell biology in the adult in response to changes in homeostasis or insults sensed in the periphery.

The Research Topic further presents three Review articles which together shed light onto how non-neuronal cells impact brain networks, synaptic function, and behavior while also examining their heterogeneity in neurodegenerative conditions like Alzheimer's disease (AD). Carrier et al. discuss how non-neuronal cells maintain and regulate structural and functional connectivity, metrics of neuronal network dynamics. Non-neuronal cells regulate neuronal populations, connectivity, energy metabolism, and also the neurovascular unit that is critical for nutrients to enter into the CNS. These functions are critical for structural and functional connectivity, but altered in schizophrenia, major depression disorder, and disorders of consciousness.

Basilico et al. describe the multitude of genetic and pharmacological strategies currently available for the depletion of microglia allowing a new avenue to investigate microglial biology. These microglial removal strategies enabled new insight into the roles that microglia play in synaptic function, learning, memory, and behavior in health and disease—highlighting the potential therapeutic use of microglial depletion in various brain pathologies involving aberrant microglia behavior.

Vu et al. outline the importance of non-neuronal cells in AD, and how single-cell (scRNAseq) and single-nucleus RNA sequencing (snRNAseq) approaches are accelerating our understanding of this disease. Non-neuronal cells were classically overlooked with AD research, but new genome wide association studies highlight several risk factors that are highly expressed in non-neuronal cells. New snRNAseq studies of postmortem tissue evaluate the disease states of non-neuronal cells, identifying new cellular states and functions during AD. Moving forward Vu et al. suggest strategies to appreciate the interconnectedness of non-neuronal cells in the context of AD.

## Author's note

M-ÈT is a Tier 2 CRC in Neurobiology of Aging and Cognition. JP is a Tier 2 Canada Research Chair in Glial Neuroimmunology. JR is a Tier 2 Canada Research Chair in Immune Regulation of Developmental Programs.

## Author contributions

JR: Writing – original draft, Writing – review & editing. M-ÈT: Writing – original draft, Writing – review & editing. JP: Writing – original draft, Writing – review & editing.

